# A 24-year integrated assessment of fluoride in surface waters of Jinhua, China: spatiotemporal distribution, risk dynamics, and future projections

**DOI:** 10.1038/s41598-026-50957-6

**Published:** 2026-05-06

**Authors:** Hongdong Lang, Yongzheng Huang, Yuling Xu

**Affiliations:** 1 Zhejiang Jinhua Ecological Environment Monitoring Center, Jinhua, 321015 China; 2 Jinhua Environmental Protection Science and Industry Federation, Jinhua, 321015 China

**Keywords:** Jinhua basin, Fluoride contamination, Long-term monitoring, Ecological risk assessment, Health risk assessment, Environmental sciences, Hydrology, Water resources

## Abstract

Fluoride contamination poses a significant global environmental and public health challenge. This study presents a comprehensive analysis of fluoride levels in the surface waters of Jinhua, China, utilizing a unique 24-year monitoring dataset (2001–2024) and a multi-faceted methodological framework. This framework integrated ecological and human health risk assessments with short-term forecasting via a hybrid ARIMA-Holt-Winters model. The multi-year mean fluoride concentration was 0.641 mg/L, 1.68-fold the regional background level but below the national drinking water standard (1.0 mg/L). Pronounced spatial and seasonal variations were observed, with downstream concentrations 137–320% higher than upstream, and dry-season levels elevated by 19.8–24.1%. Ecological risk was generally low (median risk quotient = 0.30). Health risk assessment identified children as the most vulnerable group; a pivotal shift in water source function in 2016 reduced their median hazard quotient(HQ) by approximately 97.0%. Forecasts for 2025–2029 indicate a marginal concentration increase, with risks remaining manageable. This study underscores the efficacy of source-water management and provides a transferable framework for proactive fluoride mitigation in industrial-urban regions.

**Supplementary Information:**

The online version contains supplementary material available at 10.1038/s41598-026-50957-6.

## Introduction

Fluoride is a widespread environmental contaminant that poses risks to aquatic ecosystems and human health^1,2^. In freshwater systems, chronic fluoride exposure can lead to physiological impairments^3–5^, behavioral changes, and reduced reproductive success in aquatic organisms^6^. In humans, long-term ingestion is linked to dental and skeletal fluorosis^7–12^, thyroid dysfunction^13,14^, and potential neurotoxic effects at elevated levels^15–18^.

In China, fluoride is frequently identified as a contaminant of concern in regional monitoring efforts. Previous studies have spanned multiple spatial and analytical scales, from national assessments to basin- and local city-level investigations. For instance, Ji et al.^19^ reported that 2.3% of national surface water monitoring sections exceeded quality standards, providing a macro-level perspective. At the basin scale, Zhao et al.^20^ documented associated health risks in the Yellow River basin, while at a more localized level, Dai et al.^21^ explored the geochemical behavior and potential risks of fluoride in the Xianghai Reservoir of the Songnen Plain. Methodologically, these investigations have primarily relied on field monitoring, geostatistical analysis, and deterministic health risk models. While effective for spatial assessments, this approach is inherently limited in resolving long-term temporal dynamics and source apportionment in complex environments.

Two key limitations persist in current fluoride risk assessments. First, reliance on short-term (1–3 years) and fragmented monitoring data hampers the analysis of long-term trends and cumulative effects—a critical gap for a cumulative pollutant like fluoride^22^. Second, methodological uncertainties, including non-localized exposure parameters and inadequate integration of future climate and socio-economic scenarios, can lead to risk misestimation^23,24^. These challenges are particularly acute in regions like Jinhua, where high natural background values are superimposed with intensive industrial emissions, making quantitative source apportionment difficult and understudied^25^.

Consequently, the acquisition and analysis of long-term, continuous monitoring datasets are crucial for advancing risk assessment. Such systematic long-term data are essential for uncovering latent trends, identifying delayed hazards, distinguishing natural cycles from anthropogenic influences, and validating predictive models. For assessing cumulative exposure and potential long-term ecological shifts, these datasets provide the fundamental evidence required to transition from hypothetical inference to definitive conclusions. Despite this recognized need, systematic long-term studies are scarce in regions like Jinhua, which is characterized by a dual contamination profile: a natural, fluoride-rich geology^26^ coupled with a well-developed fluorochemical industry^27^.

This “geogenic-industrial” profile distinguishes Jinhua from the primarily natural setting of the Yellow River basin^20^ or the diffuse agricultural sources of the Songnen Plain^21^. The necessity for its investigation is underscored by three distinctive features: (1) a unique geology-industry linkage where a concentrated fluorite ore zone^26^ both elevates the natural baseline and supplies local industry; (2) a point-source dominated emission structure presenting distinct regulatory challenges; and (3) heightened complexity in source apportionment, requiring methods to disentangle superimposed natural and industrial contributions.

To address these research gaps, this study leverages a unique 24-year monitoring dataset (2001–2024) from Jinhua’s major rivers. Our objectives are to: (1) quantify the spatiotemporal distribution of fluoride; (2) reconstruct historical ecological and human health risks; and (3) project future concentration trends and associated risks under different scenarios.

## Methods

### Study area

**Fig. 1:**
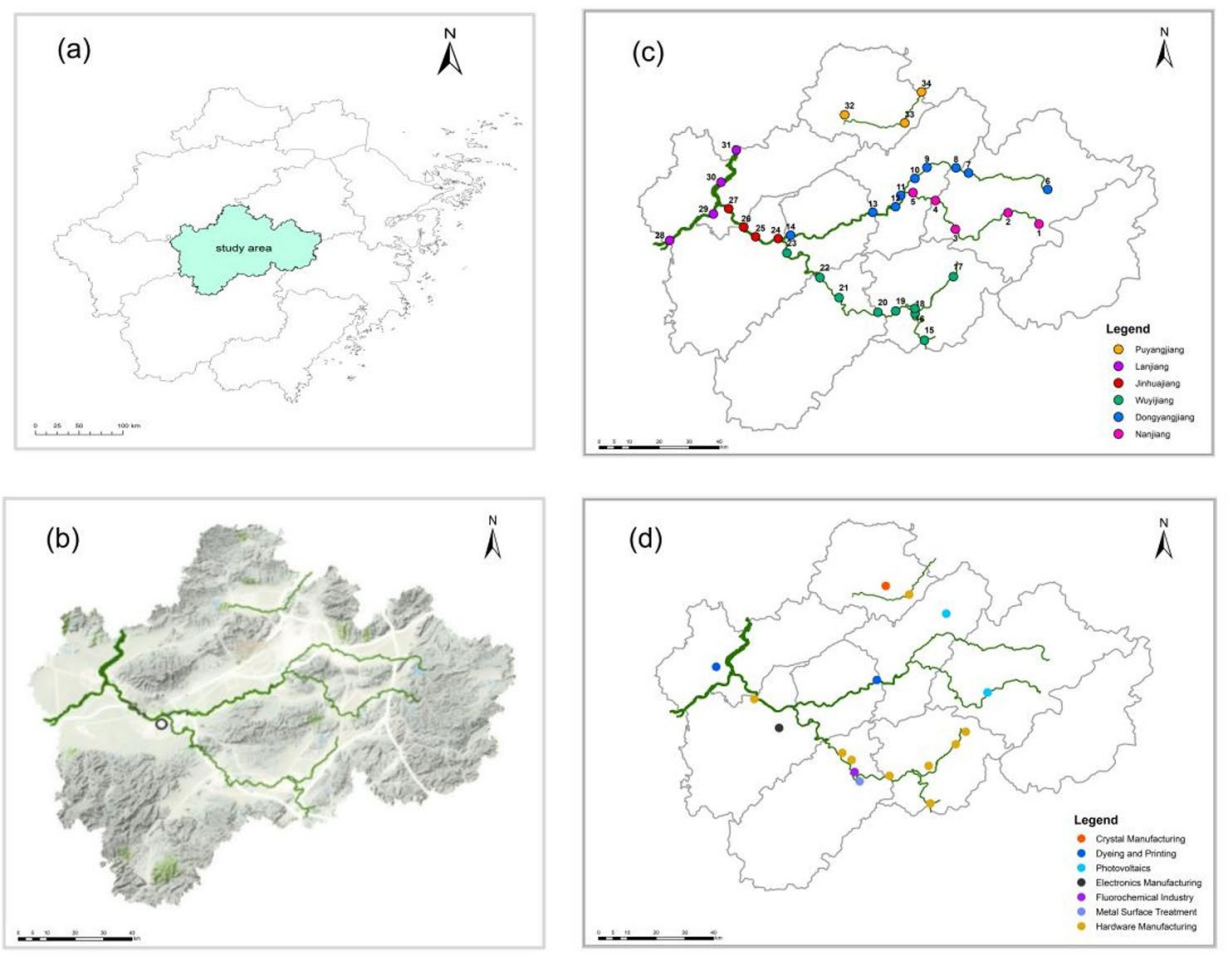
Location, geographical context, monitoring network, and industrial sources of the study area (Jinhua City). (**a**) Location within Zhejiang Province, China. (**b**) Topography and river network, highlighting the basin terrain. (**c**) Spatial distribution of the 34 numbered water-quality monitoring sections across the six major rivers. The corresponding names for site numbers 1–34 are listed in Supplementary Table S1. (**d**) Locations of major industrial point-source clusters (e.g., fluorochemical and photovoltaic manufacturing) relative to the simplified river network. All maps delineate the administrative boundary of Jinhua City. The maps were created using ArcGIS Desktop 10.8.1 (Esri, Redlands, CA, USA; https://www.esri.com).

The geographical context, topography, river networks, and key industrial clusters of the study area are illustrated in Fig. 1. Jinhua City (119°14′–120°47′ E, 28°32′–29°41′ N) is situated in central Zhejiang Province, China, within a subtropical monsoon climate zone.

Geologically, Jinhua lies within the central Zhejiang fluorite ore concentration zone, possessing abundant fluorite (CaF₂) reserves^26^. This endows the region with a naturally elevated fluoride background, as evidenced by soil fluoride levels in historical mining areas that far exceed the national average for endemic fluorosis regions^28^. Demographically, Jinhua is a major hub with an approximate population of 7.1 million (2023). Its macro-economic structure is dominated by the tertiary sector (e.g., commerce and logistics), which contributed 52.8% to the GDP in the first half of 2021^29^. However, this service-oriented profile obscures the significant environmental pressure from a specialized secondary sector, which harbors critical point sources of fluoride emissions. These include the traditional fluorochemical industry—historically developed around local fluorite resources—and the rapidly growing photovoltaic (PV) manufacturing sector, the latter having been explicitly identified in regulatory documents as a focus for fluoride emission investigations^27^.

The characteristic “riverside clustering with multi-point distribution” of these industries alongside hardware manufacturing, chemicals, and electronics poses a significant environmental challenge^25^. This challenge is exacerbated by the region’s basin topography and dense river network^26^, which collectively reduce the dilution capacity of water bodies and facilitate the transport of fluoride from both concentrated industrial point sources and diffuse natural (geogenic) inputs. The confluence of a high natural background, intensive point-source emissions, and a drainage system with limited self-purification capacity renders the aquatic environment of Jinhua particularly sensitive to additional fluoride inputs. Accordingly, official monitoring reports have confirmed a rising trend of fluoride concentrations in several rivers, establishing it as a current regulatory priority^27^.

The river network, originating from mountainous areas, is dense and features short courses, steep gradients, and rapid flow velocities. Seasonal runoff is highly uneven: the wet season spans from May to September, encompassing the plum rain period and the main flood season; the dry season occurs from November to February; and normal water levels are observed during March–April and October. The study area falls within four major basin systems, with the Qiantang River basin being dominant—covering 85.5% of the city’s area and including all six rivers investigated in this study. Within the system, the Nanjiang River is a tributary of the Dongyang River. The Dongyang River then converges with the Wuyi River to form the Jinhua River, the main stem of the basin. Downstream, the Jinhua River joins the Qujiang River, forming the Lanjiang River, a principal tributary that ultimately flows into the Qiantang River. Separately, the Puyang River enters the Qiantang River as an independent tributary. Notably, the Lanjiang River shifted from serving as a direct drinking water source to a backup source for Lanxi City in 2016.

The aquatic ecosystem is structured around phytoplankton, zoobenthos, and fish communities. Phytoplankton are primarily composed of diatoms, green algae, and cyanobacteria^30^. Zoobenthos are widely represented by pollution-tolerant taxa such as chironomid larvae, leeches, and mollusks. Fish assemblages are dominated by economically important species including carp, crucian carp, and grass carp, while native species remain relatively scarce^31^.

### Monitoring network and data collection

A monitoring network comprising 34 fixed sections was established across the six major rivers (Fig. 1). Sampling sites were strategically positioned to represent headwater (reference), upstream, mid-stream, and downstream conditions across forested, agricultural, urban, and industrial landscapes (Supplementary Table S1).

Watershed-scale fluoride concentrations were derived through a hierarchical averaging approach. Temporally, raw monthly measurements from each station were aggregated into annual means to mitigate seasonal variation. Spatially, these site-level annual values were averaged using the arithmetic mean across all monitoring stations for each year, yielding an integrated annual watershed concentration. The 24-year grand mean was calculated as the average of these annual integrated values.

The use of simple arithmetic averaging for spatial integration was based on the following considerations: (1) the monitoring network design ensured approximately equal spatial representation of major watershed compartments, minimizing bias that might otherwise necessitate area-weighted approaches; and (2) the method enhances reproducibility and comparability with other regional assessments. This approach aligns with conventional practices for watersheds with relatively uniform spatial distribution of monitoring stations^32^ and has been successfully employed in comparable long-term water quality programs, including the USGS National Water-Quality Assessment Program^[,33^.

Monthly fluoride concentration data from 2001 to 2024 were sourced from the Jinhua Surface Water Quality Monitoring Network. Water samples were collected at a depth of 0.5 m using pre-cleaned polyethylene containers and analyzed following the fluoride ion-selective electrode method (GB/T 7484 − 1987). Missing data points, accounting for fewer than 0.5% of the total, were imputed using linear interpolation^34^. The monitoring network also provided synchronous measurements of key water quality parameters, including Chemical Oxygen Demand (COD), Volatile Phenol (VPh), Ammonia Nitrogen (NH_3_–N), Total Phosphorus (TP), Total Nitrogen (TN), and Hexavalent Chromium (Cr(VI)), Cyanide, Total Petroleum Hydrocarbons(TPH), Cadmium (Cd), Copper (Cu), and Zinc (Zn).

### Ecological and health risk assessment

The potential ecological risk of fluoride was evaluated using the risk quotient (RQ) method^35^, calculated as:

1 $$\:RQ=\frac{MEC}{WQC}$$

where MEC is the measured environmental concentration (mg/L) and WQC is the chronic water quality criterion (2.0 mg/L) for protecting freshwater biota in China^36^. Risk levels were categorized as follows: RQ < 0.1 (no risk), 0.1 ≤ RQ < 1 (low risk), 1 ≤ RQ < 10 (medium risk), and RQ ≥ 10 (high risk)^37^.

The non-carcinogenic health risk from oral ingestion of fluoride was assessed in accordance with Chinese technical guidelines (WS/T 777–2021)^38^. The average daily dose (*ADD*, mg/kg·day) was calculated using the equation:

2 $$\:ADD=\frac{C\times\:IR\times\:EF\times\:ED}{BW\times\:AT}$$

Where *C* is the fluoride concentration in water (mg/L), *IR* is the ingestion rate (L/day), *EF* is the exposure frequency (days/year), *ED* is the exposure duration (years), *BW* is body weight (kg), and *AT* represents the averaging time (days).

The hazard quotient (HQ) for non-carcinogenic risk was then determined as:

3 $$\:HQ=\frac{ADD}{RfD}$$

where *RfD* is the reference dose for fluoride (0.06 mg/kg·day). An HQ threshold of 1.0 was applied; values exceeding this level indicate a potential non-carcinogenic risk of concern^39^.

To accurately reconstruct historical risk dynamics, a segment- and phase-specific exposure assessment framework was adopted, accounting for variations in public drinking water patterns across different river sections and over time.

#### Lanjiang river

A phased assessment approach was implemented to reflect the functional transition of the Lanjiang River from a direct drinking water source (2001–2015) to a conventional standby source (2016–2024). Exposure parameters for each phase were selected with the primary objective of protecting public health.

During the direct drinking water phase (2001–2015), a conservative assumption was applied, presuming no fluoride removal by centralized water treatment (i.e., treatment factor = 1). Ingestion rates (*IR*) were set at 2.4 L/day for adults and 1.5 L/day for children^40^, with an exposure frequency (*EF*) of 350 days/year^41^. The exposure duration (*ED*) was set at 15 years for adults and 12 years for children^42^ to cover their sensitive developmental periods.

After the transition to a standby source (2016–2024), the EF was adjusted to 15 days/year based on the estimated annual activation frequency. The ED was unified at 9 years for both subgroups, while IR values remained consistent with the previous phase.

#### Other rivers

For river segments not used as drinking water sources, public exposure was assumed to occur primarily through accidental ingestion during recreational activities. The *IR* was uniformly set at 0.1 L/day according to the *US EPA* Exposure Factors Handbook^43^, with an *EF* of 30 days/year representing a typical frequency for general water recreation. The *ED* was set at 24 years for adults to cover the full 24-year study period, and 12 years for children to cover their sensitive developmental periods.

Body weights (*BW*) were set at 65 kg for adults and 20 kg for children^42^. The averaging time (*AT*) was calculated as ED × 365 days^44^. A summary of all exposure parameters is provided in Supplementary Table S2.

#### Time-series analysis and forecasting

To perform short-term forecasting of fluoride concentrations in surface water, a combined modeling approach was employed, integrating both the Holt–Winters exponential smoothing method^45,46^ and the Autoregressive Integrated Moving Average (ARIMA) model^47,48^. The final forecast was generated as a weighted combination of the individual model predictions^49^, defined by:

4 $$\hat{C}\_combined = w\_ARIMA \times \hat{C}\_ARIMA + w\_HW \times \hat{C}\_HW~$$

Where *Ĉ_combined*,* Ĉ_ARIMA* and *Ĉ_HW* represent the predicted values from the combined model, the ARIMA model and the Holt-Winters model, respectively. The terms *w_ARIMA* and *w_HW* denote the combination weights assigned to the respective models, constrained by $\:w\_ARIMA+w\_HW\:=1$.

The optimal weights were determined by minimizing the sum of squared forecast errors during the validation period, calculated as:

5 $$\:w\_ARIMA=\frac{\:SSE\_HW}{SSE\_ARIMA\:+\:SSE\_HW}$$

Where *SSE_ARIMA* and *SSE_HW* represent the sum of squared errors for the ARIMA and Holt-Winters models, respectively.All analyses were conducted in Python 3.9 using the statsmodels library.

A phased multi-scenario framework was established to prospectively assess fluoride-related health risks associated with drinking water from the Lanjiang River during 2025–2029. To address uncertainties in future socioeconomic development and water management policies, three exposure scenarios were defined: Baseline, Development, and Optimization. Key exposure parameters—including exposure frequency (*EF*) and water treatment factor (*TF*)—for each scenario are detailed in Supplementary Table S3.

The Baseline scenario conservatively assumes no fluoride removal during water supply treatment (*TF* = 1.0) and maintains the current *EF.* The Development scenario projects increased water demand and more frequent use of alternative sources, leading to a higher *EF*, while TF remains 1.0. In contrast, the Optimization scenario envisions enhanced source water protection and advanced treatment, resulting in a lower *EF* and a *TF* of 0.6.

### Statistical analysis

All statistical analyses were conducted using non-parametric methods, as the Shapiro–Wilk test indicated that the data significantly deviated from a normal distribution. A significance level of *α* = 0.05 was applied to all statistical tests. Differences between two independent groups were examined using the Mann–Whitney U test. Comparisons of proportions (e.g., the fraction of samples exceeding a risk threshold or belonging to a specific risk category) were performed using the Chi-square test; for contingency tables with small expected counts, Fisher’s exact test was applied. Post-hoc analyses were conducted following significant Chi-square results to identify specific pairwise differences. Spatial and seasonal variations for continuous variables were assessed via the Kruskal–Wallis test applied to the original measurements, followed by Dunn’s test for specific pairwise comparisons. Long-term trends and potential change points in time series data were evaluated by applying the Mann–Kendall test and the Pettitt test to regional annual mean concentrations. Correlation analysis was performed using Spearman’s rank correlation coefficient, with the Bonferroni correction applied to account for multiple comparisons. Finally, scenario-based comparisons were conducted using the Friedman test, followed by post-hoc pairwise analysis.

## Results

### Spatiotemporal distribution of fluoride

**Fig. 2:**
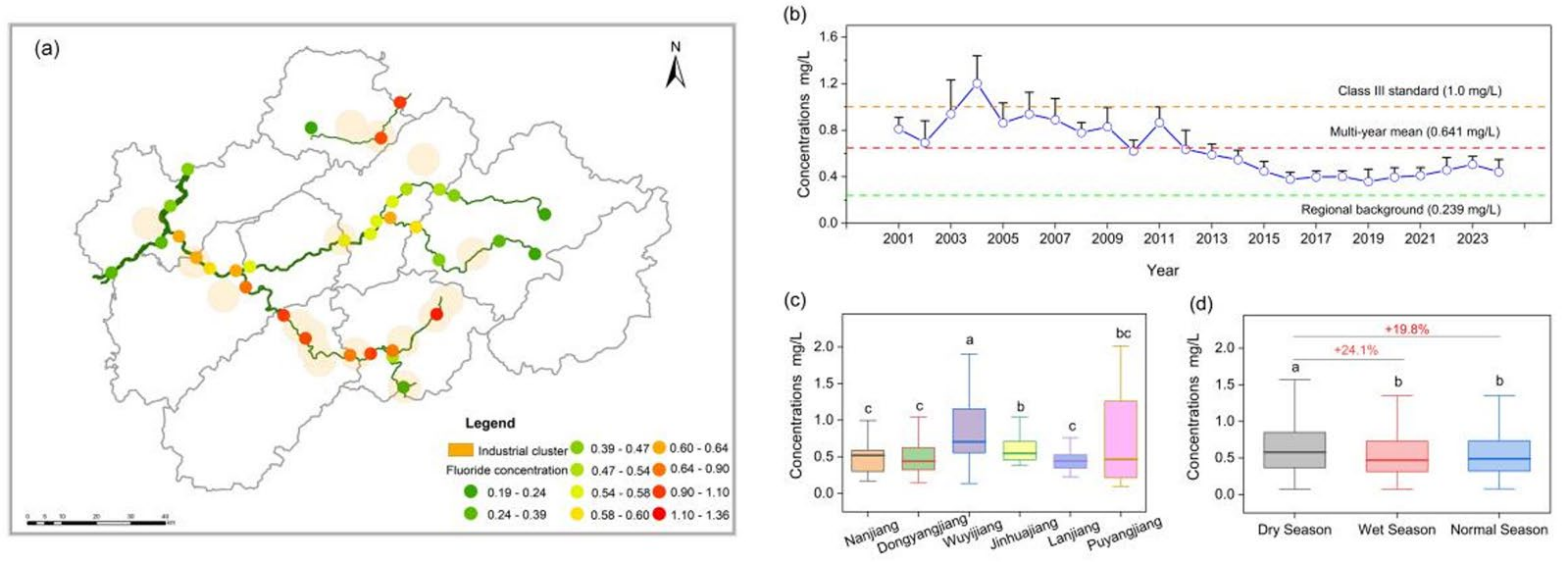
Spatiotemporal distribution of fluoride in Jinhua’s surface waters. (**a**) Spatial map of mean fluoride concentrations (2001–2024); color gradient indicates concentration level, with industrial clusters shaded in gray. The map was created using ArcGIS Desktop 10.8.1 (Esri, Redlands, CA, USA; https://www.esri.com). (**b**) Inter-annual variation, showing the multi-year mean (0.641 mg/L), regional background (0.239 mg/L), and Class III standard (1.0 mg/L). (**c**) Spatial differences across six river sections; different letters denote statistically significant differences (Kruskal–Wallis test, *p* < 0.001). (**d**) Seasonal variation; letters indicate significant differences between seasons, and percentages show the dry-season median increase. All box plots show the median, IQR, and 1.5 × IQR.

Analysis of the 24-year dataset, comprising site-year averages for each monitoring station, revealed distinct spatiotemporal patterns (Fig. 2a; Supplementary Table S4). Spatially, a clear geographical gradient was observed. Elevated concentrations were predominantly clustered in the Wuyijiang and Puyangjiang rivers (Fig. 2a), correlating with the distribution of known industrial point sources.

Inter-annually, the annual mean fluoride concentration ranged from 0.357 to 1.20 mg/L, with a multi-year average of 0.641 mg/L (Fig. 2b). This value is 1.68-fold the Zhejiang-Fujian regional background level (0.239 mg/L)^19^, yet remains below China’s Class III water quality standard (1.0 mg/L)^50^.

Spatial heterogeneity across the six rivers was statistically significant (Kruskal–Wallis test, *H*(5) = 100.50, *p* < 0.001; Fig. 2c). The Wuyijiang River exhibited the highest median concentration (0.704 mg/L). Its right-skewed distribution (mean = 0.893 mg/L) and high coefficient of variation (CV = 0.63) suggest continuous yet variable inputs, characteristic of rivers receiving concentrated industrial discharges. In contrast, the Puyangjiang River showed exceptionally high variability (CV = 0.81), indicative of intermittent pollution inputs.

Pronounced seasonal variation was also observed (Kruskal–Wallis test, *H*(2) = 97.08, *p* < 0.001; Fig. 2d; Supplementary Table S5). Dry season concentrations (median = 0.587 mg/L) were 19.8–24.1% higher than those in normal and wet seasons, underscoring a substantial dilution effect during high-flow periods.

**Fig. 3:**
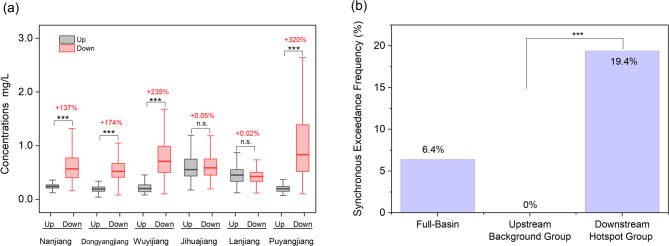
Spatial patterns of fluoride contamination. (**a**) Comparison of fluoride concentrations between upstream (grey) and downstream (red) sites in the six major rivers. The percentage indicates the median concentration increase downstream (Mann–Whitney U test; ***p* < 0.001; n.s., not significant). (**b**) Spatial contrast in synchronous exceedance frequency of fluoride, COD, and NH₃–N. Downstream hotspots (*n* = 4) showed a significantly higher frequency (19.4%) than upstream sites (0%) (*χ*² test, ***p* < 0.001).

Downstream sections of four rivers exhibited 137–320% higher median fluoride concentrations than their upstream counterparts (Mann–Whitney U tests, all *p* < 0.001; Fig. 3a). Upstream sites, representing the regional background, consistently showed low levels (median: 0.21 mg/L). This marked downstream increase against a low-background baseline strongly suggests cumulative point-source inputs rather than regional geological enrichment. This spatial pattern was corroborated by the analysis of synchronous high-concentration events (Fig. 3b). The basin-wide frequency of synchronous exceedance of fluoride, COD, and NH_3_–N was 6.4%. In stark contrast, the frequency reached 19.4% in the identified downstream hotspots but was 0% upstream (*χ²*(1) = 247.87, *p* < 0.001; for station classification see Supplementary Table S6). At a representative hotspot (‘Wuyijiang-Shiya’), over 30% of samples exhibited this triple co-exceedance. The concurrence of elevated median concentrations and highly localized compound pollution events pinpoints areas receiving industrial discharges as contamination foci.

By contrast, no significant longitudinal gradient is detected in the Jinhuajiang and Lanjiang Rivers (Fig. 3a). This indicates a fundamentally different longitudinal dynamic compared to the tributaries.

### Correlation with industrial pollution indicators

**Fig. 4:**
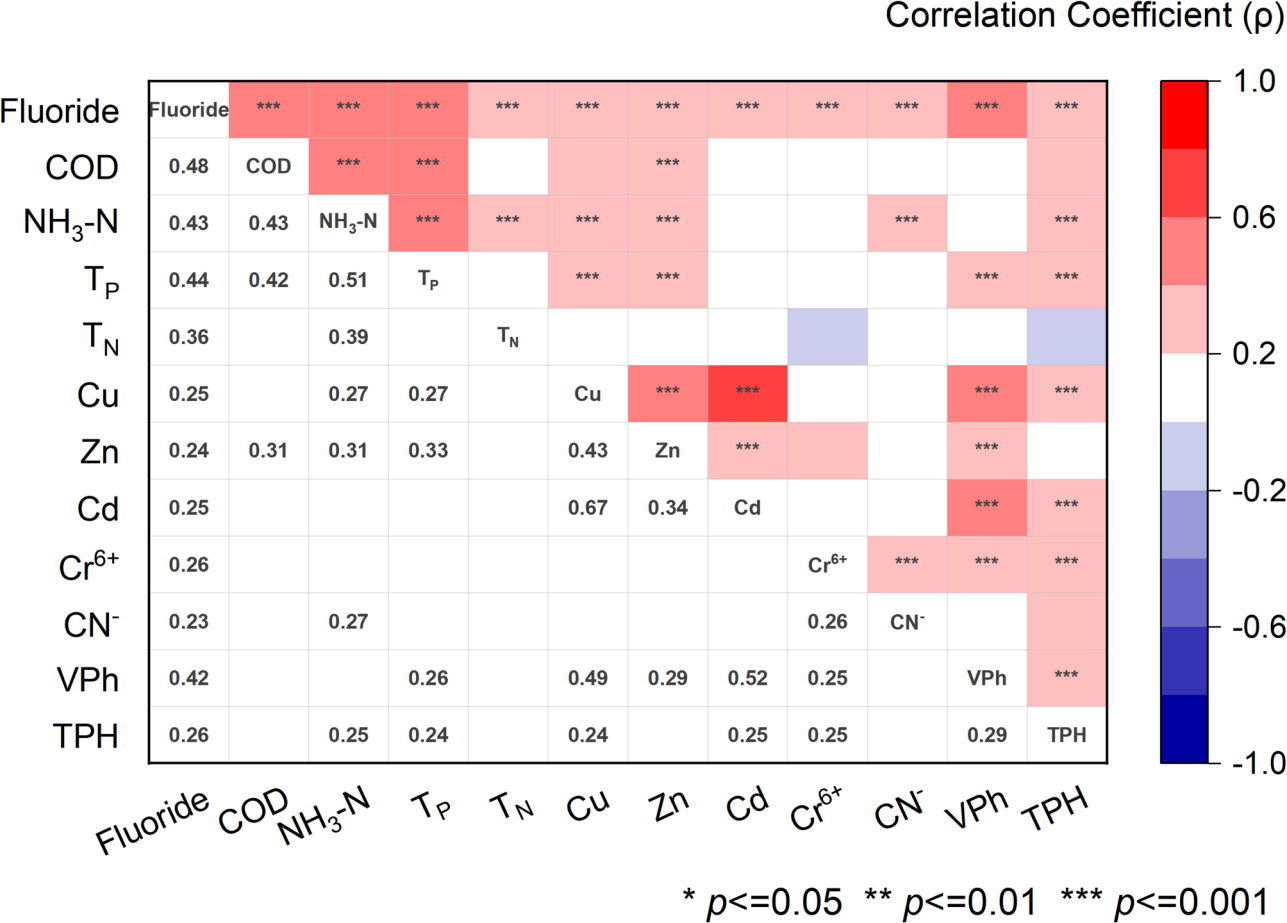
Correlation between fluoride and industrial pollution indicators. Heatmap displays statistically significant Spearman’s rank correlation coefficients (*ρ*; all *p* < 0.001). Color intensity corresponds to the strength of the correlation (see color bar). COD, chemical oxygen demand; VPh, volatile phenol; NH_3_–N, ammonia nitrogen; TP, total phosphorus; TN, total nitrogen; Cu, copper; Zn, zinc; Cd, cadmium; Cr(VI), hexavalent chromium; CN^−^, cyanide; TPH, total petroleum hydrocarbons.

Fluoride concentrations showed significant positive correlations with multiple industrial pollutants (Spearman’s *ρ*, all *p* < 0.001; Fig. 4; Supplementary Table S7). The strength of most statistically significant correlations was weak to moderate^51^. Specifically, moderate correlations (0.40 ≤ |*ρ*| < 0.60) were observed with conventional indicators of organic and nutrient pollution, including COD (*ρ* = 0.48), TP (*ρ* = 0.44), NH_3_–N (*ρ* = 0.43), and VPh (*ρ* = 0.42). Weaker but significant correlations (0.20 ≤ |*ρ*| < 0.40) were found with specific toxic substances, such as CN^-^ (*ρ* = 0.23), TPH (*ρ* = 0.26), Cd (*ρ* = 0.25), Cu (*ρ* = 0.25), and Zn (*ρ* = 0.24), as well as with TN (*ρ* = 0.36).

The persistent co-occurrence and correlation between fluoride and this suite of industrial pollutants suggest a shared origin, likely composite industrial wastewater. Notably, the maximum correlation strength observed was moderate (*|ρ|* < 0.6), which is common in environmental field studies^52^. This pattern reflects the reality that fluoride concentrations in surface waters are influenced by a multitude of interacting natural and anthropogenic factors, leading to complex, non-univariate relationships that are not fully captured by pairwise correlation alone.

### Ecological risk assessment

**Fig. 5:**
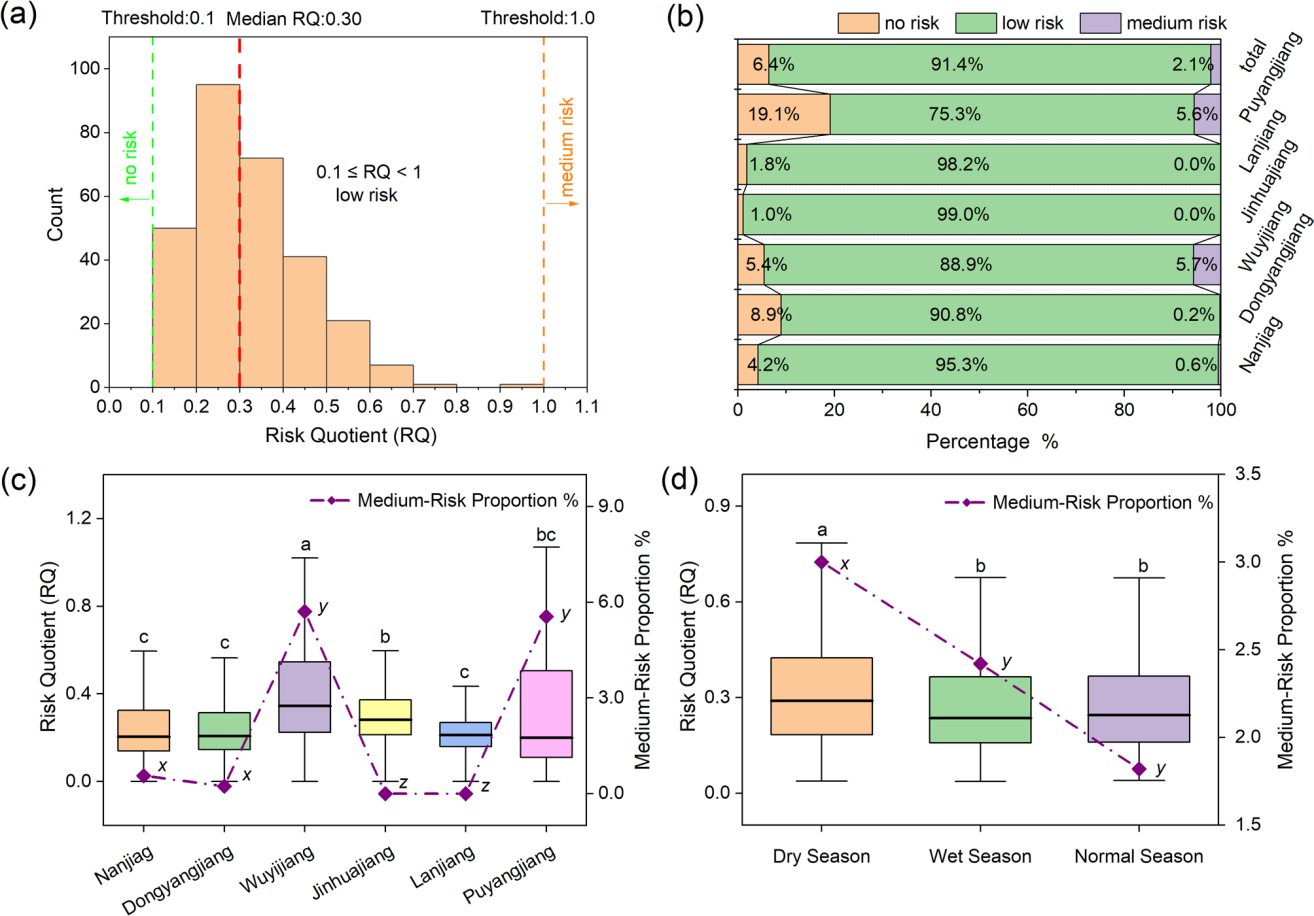
Ecological risk assessment of fluoride in surface waters. (**a**) Frequency distribution of the risk quotient (RQ). Dashed lines indicate low-risk (RQ = 0.1), medium-risk (RQ = 1.0) thresholds, and the median (0.30). (**b**) Spatial distribution of ecological risk levels. (**c**,**d**) Spatial and seasonal variations in median RQ (box plots) and the proportion of medium-risk samples (RQ ≥ 1.0; point-line plots). Different letters denote statistically significant differences (Kruskal–Wallis test with Dunn’s post hoc for RQ; Chi-square test for proportions; *p* < 0.05). Box plots show median, IQR, and 1.5 × IQR.

The overall ecological risk of fluoride in the study area was low, with a median risk quotient (RQ) of 0.30 (Fig. 5a). Spatial analysis, however, revealed significant differences among river sections (Kruskal–Wallis test with Dunn’s post hoc comparison, *p* < 0.05; Fig. 5b, c). While low-level ecological concern (RQ ≥ 0.1) was widespread (affecting 91.4% of samples), elevated risks were concentrated in specific areas. Notably, medium-risk samples (RQ ≥ 1.0) were far more frequent in the Wuyijiang (5.7%) and Puyangjiang (5.6%) Rivers. In contrast, they were exceedingly rare (< 1.0%) or entirely absent in all other rivers. Seasonally, the median RQ during the dry season was significantly higher than during the wet and normal seasons (Kruskal–Wallis test with Dunn’s post hoc comparison, *p* < 0.05; Fig. 5d). The proportion of medium-risk samples also varied significantly both spatially and seasonally (Chi-square test with post hoc analysis, *p* < 0.05; Fig. 5c, d), with higher proportions consistently observed in the identified high-risk rivers and during the dry season.

### Health risk assessment

**Fig. 6:**
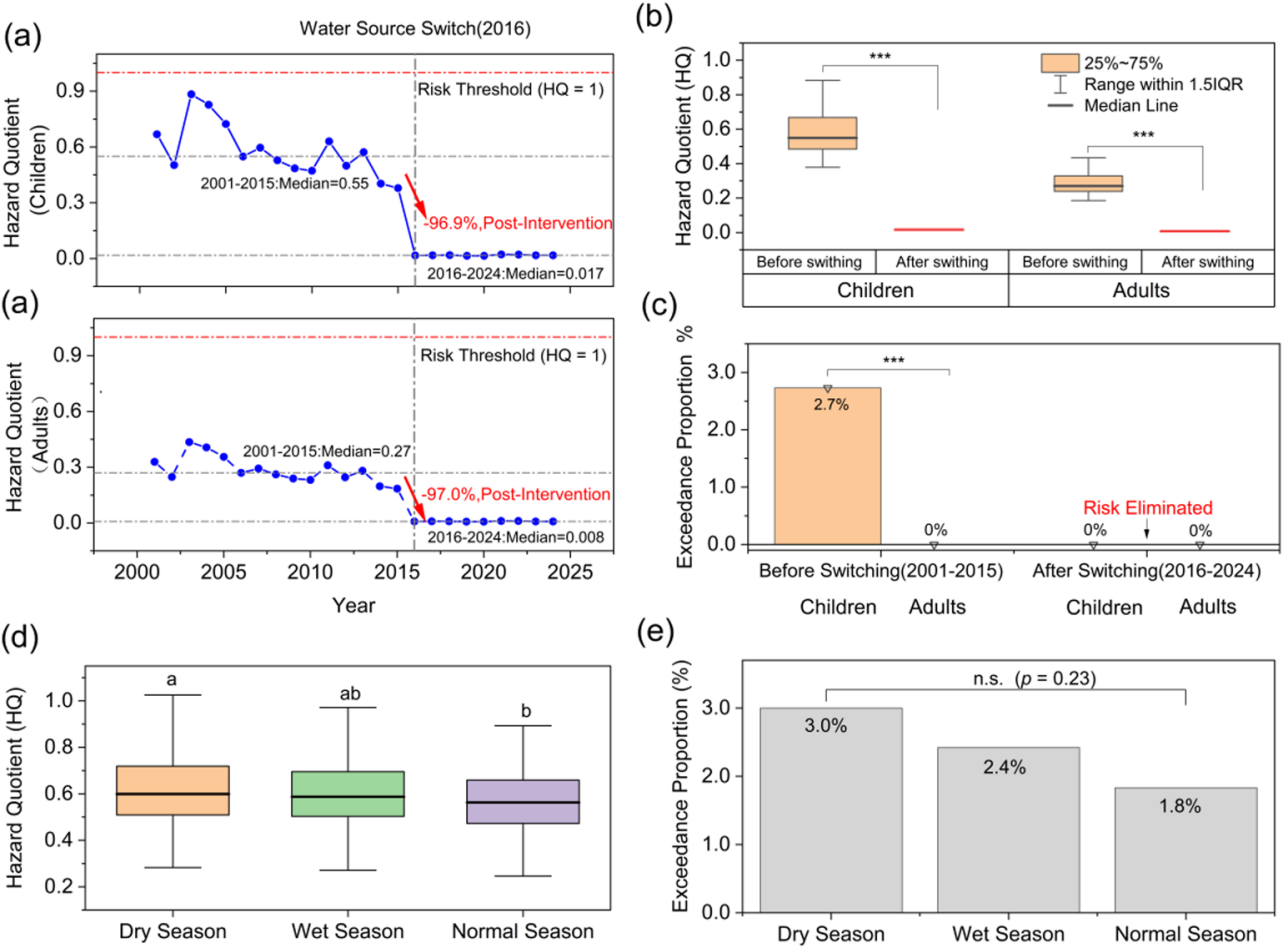
Reduction in fluoride-related health risk following the 2016 drinking-water source transition. (**a**) Time series of hazard quotient (HQ) for children and adults; the dashed line marks the intervention. Arrows indicate an ~ 97.0% reduction in median HQ. (**b**) Comparison of HQ distributions before and after intervention (Mann–Whitney U test; ***p* < 0.001). (**c**) Proportion of samples exceeding the risk threshold (HQ > 1) before and after intervention; *** indicates a significant difference between children and adults pre-2016 (Fisher’s exact test, *p* < 0.001). (d, e) Seasonal variation in HQ (**d**) and in the proportion of HQ > 1 samples (**e**) for children during the pre-intervention period (letters denote significant differences; n.s., not significant). Box plots show median, IQR and 1.5 × IQR; the horizontal dashed line in (**a**,**b**) marks HQ = 1.

The health risks associated with fluoride exposure were evaluated under distinct scenarios, with a focus on the impact of a major water management intervention. The results are summarized in Fig. 6.

#### Temporal trend following source-water shift

A pronounced decrease in risk was observed following the transition of the Lanjiang River from a direct drinking water source (2001–2015) to a backup source in 2016. During the pre-intervention period, the median hazard quotient (HQ) was 0.55 for children and 0.27 for adults. After 2016, the median HQ values declined to 0.017 and 0.008, respectively, representing a reduction of approximately 97.0% (Mann-Whitney U test, U = 135, *p* < 0.001; Fig. 6a, b).

#### Risk disparity between demographic groups

Prior to the intervention, the absolute proportion of samples exceeding the risk threshold was low. Nevertheless, a statistically significant difference was found between children and adults (Fisher’s exact test, *p* < 0.001; Fig. 6b, c): 2.7% of samples posed a potential risk (HQ > 1) to children under the direct consumption scenario, whereas no exceedances were recorded for adults. Following the intervention, no exceedances were observed for either group.

#### Seasonal variation in pre-intervention risk

Analysis of the pre-intervention period revealed significant seasonal variation in HQ for children, with significant differences between dry and normal seasons (Kruskal–Wallis test with Dunn’s post hoc comparison, *p* < 0.05; Fig. 6d). In contrast, the proportion of samples exceeding the risk threshold (HQ > 1) for children did not vary significantly by season (Dry: 3.0%; Wet: 2.4%; Normal: 1.8%; Chi-square test, *p* = 0.23; Fig. 6e).

#### Risk under recreational exposure

For all other rivers, exposure was assessed under a recreational scenario (incidental ingestion during swimming/fishing), which entails low ingestion rates and exposure frequency. Within this specific exposure framework, all calculated HQ values remained below 1.0, with median values of 0.0045 for children and 0.0014 for adults. Thus, the assessment indicates that fluoride concentrations in these rivers did not lead to appreciable health risks under the modeled recreational exposure assumptions.

### Historical trends and risk dynamics

#### Trends in fluoride and ecological risk

**Fig. 7:**
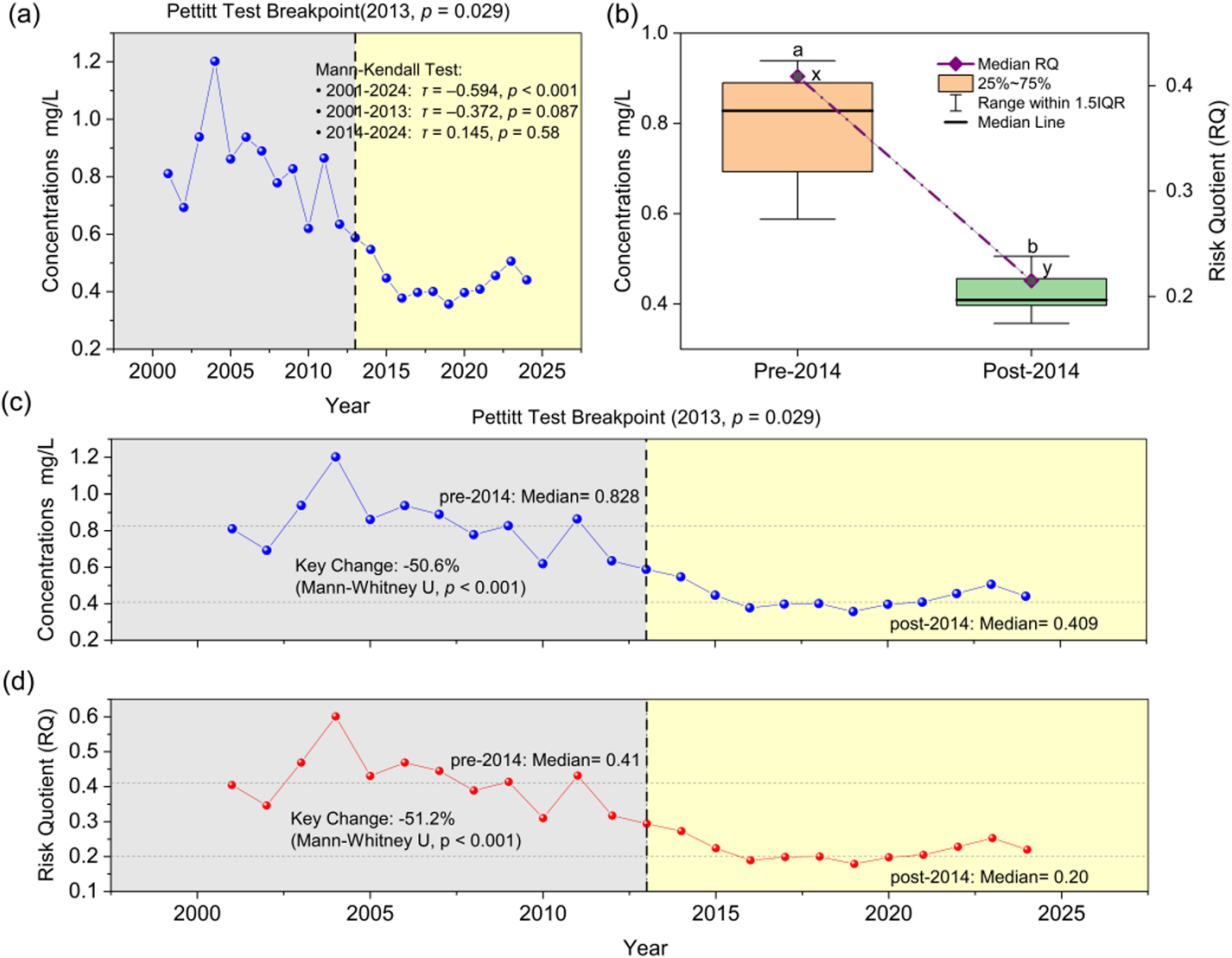
Long-term trends in fluoride concentrations and ecological risk (2001–2024). (**a**) Temporal trend of annual fluoride concentrations. A statistically significant breakpoint in 2013 (Pettitt test, *p* = 0.029) separates the series into two phases, with an overall significant decreasing trend (Mann-Kendall test, *p* < 0.001). (**b**) Comparison of fluoride concentrations (box plots) and median ecological risk quotient (RQ; point-line plots) between the two phases. (**c**,**d**) Aligned long-term trends of fluoride concentrations (**c**) and the ecological risk quotient (RQ). (**d**) The RQ was calculated as concentration divided by the water quality criterion (WQC). The vertical dashed line indicates the 2013 breakpoint. Box plots show median, IQR, and 1.5 × IQR. Shaded backgrounds in (**a**,**d**) mark the pre- and post-breakpoint phases.

Analysis of the basin-wide monitoring dataset (2001–2024) revealed a statistically significant decreasing trend in fluoride concentrations over the entire period (Mann-Kendall test, *τ* = − 0.594, *Z* = − 4.05, *p* < 0.001; Fig. 7a; Supplementary Table S8). The Pettitt test identified 2013 as a significant breakpoint (*p* = 0.029). Although no significant monotonic trend was detected within the resulting two sub-periods (pre- and post-2014), a substantial shift in concentration levels occurred: median fluoride concentrations decreased by 50.6% from 0.828 mg/L (pre-2014) to 0.409 mg/L (post-2014) (Mann-Whitney U test, *U* = 10, *p* < 0.001; Fig. 7b, c).

Given the deterministic calculation of the ecological risk quotient (RQ = Concentration / WQC), its long-term dynamics exactly mirrored the concentration trends (Fig. 7c, d). Consequently, the median RQ also decreased significantly by 51.2% after 2013 (Mann-Whitney U test, *U* = 8, *p* < 0.001; Fig. 7b, d). This synchronous, stepwise decline in both concentration and ecological risk is temporally associated with the implementation of regional water quality management policies.

#### Fluoride and health risk evolution in Lanjiang river

**Fig. 8:**
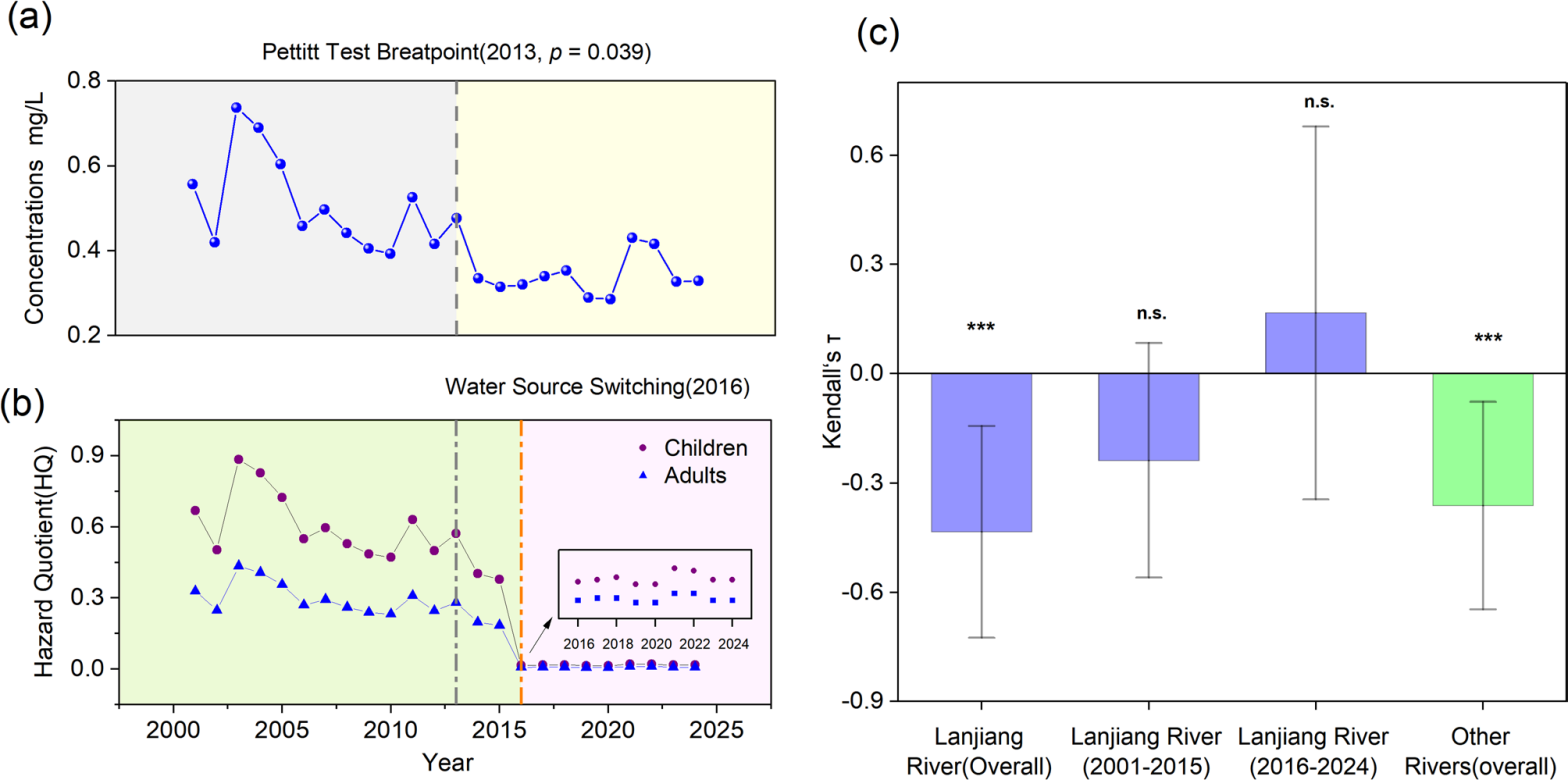
Spatio-temporal patterns of health risk evolution from fluoride. (**a**) Temporal trend of annual fluoride concentrations in the Lanjiang River, showing a significant breakpoint in 2013 (Pettitt test, *p* = 0.039). (**b**) Corresponding trend in the hazard quotient (HQ) for children and adults, with the 2016 drinking-water source switch marked. (**c**) Trend strength (Kendall’s *τ*) and statistical significance of fluoride risk reduction in the Lanjiang River versus other rives. Error bars represent 95% confidence intervals. Significance levels: ***p < 0.001.

The Lanjiang River, due to its historical role as a drinking water source, exhibited a distinct trajectory. Its long-term fluoride concentration trend was not statistically significant overall (Mann-Kendall test, *p* = 0.079; Fig. 8a; Supplementary Table S9). However, the Pettitt test identified a significant breakpoint in 2013 (*p* = 0.039), partitioning the data into two phases. Although subsequent Mann-Kendall tests showed no significant monotonic trend within either phase, a Mann-Whitney U test confirmed a significant downward shift in concentration levels after 2013 (*p* < 0.001). The median concentration decreased by 31.6%, from 0.497 mg/L to 0.340 mg/L.

In contrast, the associated health risk showed a significant decreasing trend (Mann-Kendall test, *τ* = − 0.434, *p* = 0.004; Fig. 8b; Supplementary Table S10). A Pettitt test on the health risk series identified a further significant breakpoint in 2015 (*p* < 0.01), supporting a three-phase dynamic linked to water use changes. In Phase 1 (2001–2013), when the river served as a primary drinking water source, hazard quotients (HQ) remained consistently elevated, with median values of 0.57 for children and 0.28 for adults. Phase 2 (2014–2015) represented a transitional period, showing moderately lower median HQ values of 0.40 for children and 0.20 for adults. Phase 3 (2016–2024) began after its reassignment as a backup source, during which the median HQ dropped sharply and stabilized at very low levels of 0.017 for children and 0.008 for adults.

#### Basin comparison of risk reduction

The drivers of risk reduction differed markedly between the Lanjiang River and other basins (Fig. 8c; Supplementary Table S10). In the Lanjiang River, the significant overall decline in health risk was primarily driven by the abrupt drop after the 2016 water source shift, as sub-trends before and after this intervention were non-significant. Conversely, other rivers exhibited a consistent and significant continuous decreasing trend over the entire study period (*τ* = − 0.362, *p* = 0.014), indicative of a more gradual mitigation process.

### Future projections and scenario analysis

**Fig. 9:**
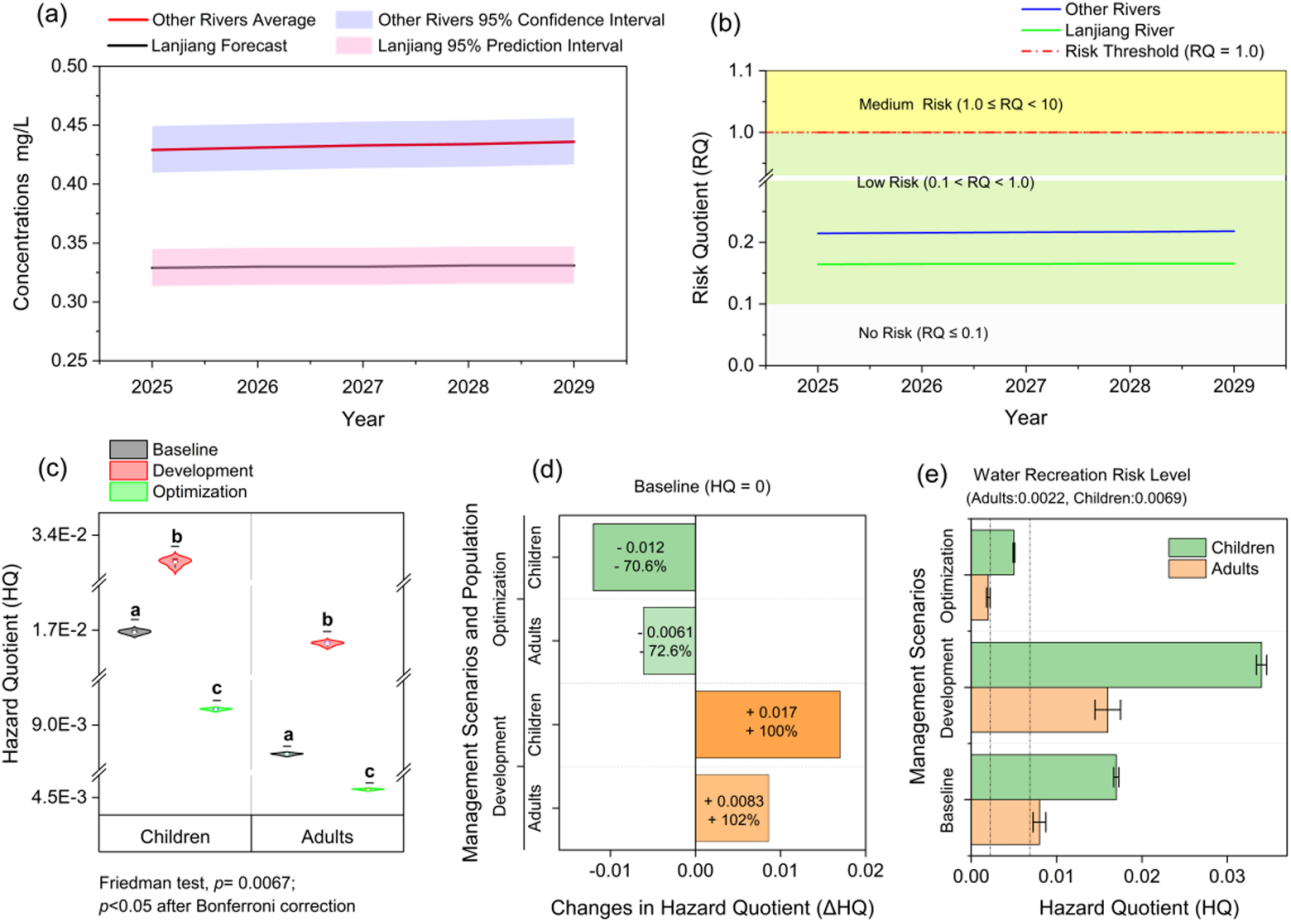
Projected trends in fluoride concentrations and associated risks (2025–2029). (**a**) Forecasted fluoride concentrations for the Lanjiang River and the average of other rivers (shaded areas: 95% confidence intervals). (**b**) Projected ecological risk quotient (RQ) for the Lanjiang River (RQ ≈ 0.16–0.17) and other rivers (RQ ≈ 0.21–0.22). The dashed line marks the medium-risk threshold (RQ = 1.0); background shading indicates risk categories. (**c**) Comparison of health risk (HQ) distributions for children and adults under three management scenarios (Development, Business-as-Usual, Optimization). Different letters denote statistically significant differences in median HQ (adjusted *p* < 0.05). (**d**) Effectiveness of interventions: percentage change in median HQ relative to the baseline. (**e**) Median health risk (HQ) across scenarios for children and adults; whiskers show 1.5 × IQR (magnified 10×). Dashed lines indicate background recreational exposure; all values remain below the safety threshold (HQ = 1.0).

Robust time-series modeling (Supplementary Table S11) projected a gradual increase in fluoride concentrations in Jinhua surface waters during 2025–2029. Concentrations stabilized at 0.329–0.331 mg/L in the Lanjiang River and 0.429–0.436 mg/L in other rivers, remaining well below the 1.0 mg/L water quality standard (Fig. 9a; Supplementary Table S12). Corresponding ecological risks remained low. The projected risk quotients (RQ) are expected to stabilize between 0.16 and 0.17 for the Lanjiang River and 0.21–0.22 for other rivers, remaining below the established low-risk threshold (RQ = 1.0; Fig. 9b).

A multi-scenario health risk forecast for the Lanjiang River as a backup drinking water source revealed substantial variation across exposure scenarios (Supplementary Table S3). The Friedman test confirmed statistically significant differences among scenarios (*χ*²(2) = 10.04, *p* = 0.0067). The median hazard quotient (HQ) showed a clear ordinal pattern: the development scenario (increased backup frequency) posed the highest risk (median HQ = 0.034 for children, 0.017 for adults), followed by the business-as-usual baseline (median HQ = 0.017 for children, 0.0084 for adults), while the optimization scenario (reduced frequency with advanced treatment) showed the lowest risk (median HQ = 0.0047 for children, 0.0023 for adults). Relative to the baseline, the optimization scenario reduced the median HQ by 70.6% for children and by 72.6% for adults. In contrast, the development scenario increased the median HQ by 100% for children and by 102% for adults, highlighting the pronounced influence of management pathways (Fig. 9c, d).

Notably, under the optimization scenario, the median health risk(HQ) decreased to 0.0023 for adults and 0.0048 for children—levels approaching background recreational exposure (adults: HQ = 0.0022; children: HQ = 0.0069; Fig. 9e). These results demonstrate that proactive interventions, including advanced water treatment and source water protection, can effectively mitigate fluoride-related health risks even amid rising environmental concentrations.

## Discussion

This study presents a multi-decadal analysis of fluoride contamination within the Jinhua river network. The following discussion interprets the key findings, elucidates the underlying mechanisms, and explores their implications for environmental science and management.

### Sources and dynamics of fluoride contamination

#### Spatiotemporal patterns

Spatially, elevated fluoride levels in the Wuyijiang and Puyangjiang rivers align with known industrial clusters (Fig. 2a, c). The observed concentration patterns provide diagnostic signatures of point-source influence. In the Wuyijiang River, the right-skewed distribution and high coefficient of variation (CV = 0.63) are characteristic of systems receiving continuous yet variable discharges from concentrated industrial activities. Conversely, the exceptionally high variability (CV = 0.81) in the Puyangjiang River suggests a regime dominated by intermittent, episodic inputs, such as from irregular industrial releases. These distinct statistical fingerprints, coinciding with industrial zones, substantiate that point-source discharges are a primary control over the spatial heterogeneity.

Temporally, the 19.8–24.1% higher concentrations in the dry season highlight a strong dilution effect during high-flow periods. The absence of a significant difference between normal and wet seasons suggests non-point source runoff may partially offset dilution during rainfall events^53,54^^55^, potentially through the washoff of pollutants from urban and industrial areas or the re-suspension of sediment-bound contaminants^56^.

#### Longitudinal gradients

A key finding is the divergent longitudinal behavior between tributaries and mainstem rivers. Four tributaries functioned as “source conduits,” exhibiting significant downstream concentration increases (137–320%; Fig. 3a). This pattern confirms cumulative inputs from localized point sources and aligns with observations of point-source dominated systems^57^.

In contrast, the Jinhuajiang and Lanjiang Rivers (mainstems) showed no significant longitudinal gradient, shifting to a “transport-dilution-attenuation” regime. This divergence is explained by: (i) greater dilution capacity in mainstems due to higher base flow^58,59^, (ii) potential in-stream removal processes such as adsorption to suspended solids or sediments^60^^61,62^, and (iii) flow regulation by reservoirs which increase hydraulic retention time and can reset concentration gradients^58,63^. This contrast underscores the need to prioritize fluoride mitigation in tributaries functioning as “source conduits.”

#### Source evidence and attribution

The attribution of elevated fluoride to industrial point sources is supported by converging lines of evidence. First, fluoride shows a distinct statistical co-occurrence fingerprint with conventional industrial pollutants (COD, NH_3_–N, volatile phenols; Spearman’s *ρ* ≈ 0.40–0.50), indicating a shared origin from composite effluent rather than independent pathways. Second, strong spatial co-localization and magnitude contrast geographically focus the contamination. Downstream hotspots for fluoride and its correlated pollutants precisely coincide, consistently proximate to documented industrial parks. Median concentrations in these hotspots were 137–320% above low upstream background levels (median: 0.21 mg/L). The frequency of synchronous high-concentration events for these parameters reached 19.4% in impacted zones, contrasting with 0% upstream, underscoring a common, point-source origin. Third, this co-release pattern finds mechanistic and institutional plausibility. The specific pollutant association aligns with documented emission profiles of key local industries (e.g., photovoltaic, fluorine-based material manufacturing) and is corroborated by institutional records such as environmental permits^27,64,65^.

Moreover, alternative drivers are inconsistent with the evidence. The persistently low upstream background contradicts regional geology as a primary source. Furthermore, the patterns—persistent dry-season peaks, right-skewed distributions, and high spatial variability at hotspots—contrast starkly with the diffuse signal expected from agricultural runoff or natural leaching.

Collectively, the integration of statistical fingerprinting, spatial co-localization, and mechanistic plausibility forms a coherent evidence triad. This synthesis robustly identifies composite industrial discharges as the principal cause of the region’s fluoride contamination.

#### Historical trends and mechanisms of risk evolution

Long-term trend analysis followed the principle of detection first, association second. Pettitt’s test identified a statistically significant (*p* < 0.05) regime shift in fluoride concentration around 2013/2014, marked by an abrupt 50.6% decline in the median level. This data-derived breakpoint (2013), which precedes the launch of the comprehensive ‘Five-Water Co-governance’ policy in 2014, suggests that the policy helped to consolidate and sustain the initial improvement.

Applying the same detection-first approach to population health risk (HQ) in the Lanjiang River revealed a separate, sharper breakpoint—a ~ 97.0% decline centered on 2015/2016. This risk breakpoint aligns precisely with the discrete engineering intervention of switching to a protected municipal water supply. The notable temporal decoupling, in which water quality improvement (2013/2014) preceded drastic risk reduction (2015/2016), delivers a pivotal insight: population health risk is governed not merely by ambient concentration, but fundamentally by human exposure pathways. This distinction is critical for risk management prioritization.

Spatially, two divergent improvement pathways emerged: the Lanjiang River showed a sharp, step-change decline tied exclusively to infrastructure intervention, whereas other rivers exhibited a steady, monotonic decline consistent with sustained basin-wide pollution control. This dichotomy underscores that management strategies must be tailored to the dominant driver in each context—whether rapid exposure interception or gradual source control.

In summary, this temporally resolved analysis provides empirical evidence for a sequential causal chain: policy reinforcement likely stabilized water quality gains, but the order-of-magnitude collapse in population risk was achieved primarily through engineered exposure pathway control. The Lanjiang River case thus offers a compelling model, demonstrating that integrating targeted infrastructure with source remediation can yield disproportionate public health benefits, a insight widely applicable to similar contamination settings.

### Ecological and health risk implications

#### Spatially prioritized ecological risks

Consistent with their identification as primary fluoride point-source hotspots, the Wuyijiang and Puyangjiang Rivers also concentrate ecological risk. Assessment indicated generally low basin-risk (median risk quotient, RQ = 0.30) to aquatic ecosystems under current conditions, yet these tributaries exhibit a statistically significant higher frequency of medium-risk samples (RQ ≥ 1.0; 5–6% of local samples) compared to other rivers. Critically, exceedances form spatial clusters downstream of industrial complexes. Over 90% of the basin’s medium-risk samples are concentrated here, and risk is heightened during low-flow periods. Therefore, management demands spatially and temporally targeted strategies.

### Health risk vulnerability, intervention the decisive role of exposure pathways

Human health risk assessment under the direct water consumption scenario identified children as the most vulnerable demographic, a finding with critical implications for risk management.

#### Demographic vulnerability and its significance

A statistically significant disparity in risk was confirmed, with children’s hazard quotients approximately double those of adults—a difference mechanistically explained by physiological factors including lower body weight and higher water intake. Although the absolute proportion of samples exceeding the risk threshold was low (2.7% for children vs. 0% for adults; Fig. 6c), this disparity holds substantial public health relevance. Within a precautionary framework, the statistically significant concentration of risk within the most sensitive population subgroup necessitates targeted attention, justifying its emphasis as a key assessment outcome and aligning with patterns observed in other fluoride-affected regions^66^.

#### Seasonal dynamics and the efficacy of intervention

A nuanced analysis distinguished between seasonal concentration fluctuations and actual risk exceedance. While HQ values were statistically higher in the dry season, the rate of threshold exceedance did not vary significantly by season (Fig. 6d, e), indicating that measurable concentration increases seldom translated into a higher frequency of critical risk events.

The 2016 transition of the Lanjiang River to a backup water source provided a decisive solution, reducing the fluoride hazard quotient(HQ) by approximately 97.0% and eliminating excess risk for children. This intervention starkly demonstrates that identified vulnerabilities are addressable through engineered infrastructure that severs the primary exposure pathway. It underscores the exceptional efficacy of source-water management and argues for risk strategies that prioritize protecting vulnerable subgroups through targeted interventions.

#### The critical role of exposure scenarios

For rivers not serving as drinking water sources, it is important to recognize that the derived risk conclusions are fundamentally contingent on the defined exposure scenario. Under the conservative parameters of a realistic recreational exposure scenario, the calculated hazard quotient (HQ) remained well below 1.0 for all such rivers, indicating negligible risk. This outcome is thus a direct consequence of the low-exposure assumptions intrinsic to this scenario.

Consequently, the primary value of this analysis lies not in the absolute risk estimate for any single scenario, but in the consistent comparative framework it establishes. Applying the same conservative recreational baseline across all non-potable rivers creates a valid internal standard. The analytical power of this approach is clearly demonstrated when this baseline is juxtaposed with the pre-2016 direct drinking water scenario for the Lanjiang River. This comparison reveals an exposure-driven hazard increase of several orders of magnitude. Thus, the stark, internally consistent contrast serves as a robust and objective demonstration of the profound population health benefit achieved by switching to a protected water supply—an insight that remains valid independent of the specific recreational parameters. Within any given scenario, relative risk comparisons (e.g., children vs. adults) remain equally robust.

### Uncertainty, limitations, and future perspectives

Several limitations should be considered when interpreting these findings.

#### Parameter and model uncertainty

The deterministic hazard quotient (HQ) calculations are inherently sensitive to the selection of exposure parameters (e.g., ingestion rate, exposure frequency) and toxicological reference doses (*RfD*). Our adoption of conservative assumptions—including the use of upper-percentile intake rates and the omission of water treatment removal efficiencies in historical scenarios—aligns with a precautionary approach. While these choices influence the absolute magnitude of risk estimates, they ensure the robustness of the core comparative findings (e.g., the differential vulnerability of children, the pronounced risk reduction post-2016), as the same parameters were applied consistently across comparisons. Similarly, the ecological risk quotient (RQ) method serves as a screening-level tool useful for prioritization but does not account for complex mixture toxicity or potential sub-lethal biological effects.

#### Scenario and structural uncertainty

All human health risk conclusions are explicitly scenario-dependent. The assessments for non-potable rivers are conditional upon the specific, low-exposure recreational scenario modeled, and extrapolation to other use patterns is not warranted. Furthermore, the models did not fully incorporate dynamic external drivers such as evolving industrial emissions or long-term climate change effects, which represents a structural limitation for projecting future trends.

#### Data representativeness and confounding

The reliance on annual averaged concentration data, while necessary for long-term trend analysis, may obscure short-term, high-concentration pulses that could pose acute risks. Spatial characterization is subject to uncertainty introduced by interpolation between fixed monitoring stations. Perhaps most critically, while the observed temporal associations between fluoride declines and policy interventions are compelling, the influence of unmeasured or unmodeled confounding factors cannot be entirely ruled out.

Collectively, these uncertainties define the boundary conditions and interpretive limits of our study rather than invalidate its core insights. The principal value of this work lies in the identification of clear relative risk patterns, spatial priorities, and long-term trends that are directly informative for environmental management.

To advance beyond these limitations, future research should aim to develop a more integrated assessment framework. This includes: (1) incorporating localized exposure parameters and water treatment efficiency data to refine risk characterization; (2) expanding predictive models to include dynamic socioeconomic and climate scenarios; and (3) complementing chemical benchmarking with biomonitoring programs, epidemiological investigations, and the assessment of mixture toxicity. Such efforts would substantially enhance the realism and utility of risk assessments for environmental and public health decision-making.

## Conclusion

This 24-year integrated assessment provides critical insights for environmental and public health policy. First, despite currently low overall risks, fluoride contamination displays substantial spatiotemporal heterogeneity, This pattern is driven primarily by industrial point-source discharges, requiring targeted monitoring strategies. Second, public health risk is critically pathway-dependent. The 2016 source-water intervention, which achieved a ~ 97.0% risk reduction by switching to a treated supply, demonstrates the decisive effectiveness of proactive infrastructure management and identifies children as the most vulnerable subgroup, offering a transferable model for regions facing similar challenges. Third, long-term data reveal that improvements in water quality and population health can be decoupled; a ~ 51.0% decline in fluoride concentrations followed earlier policy actions, whereas the sharper health risk decline lagged until the 2016 engineering measure. scenario-based projections confirm that future risks can be effectively controlled through anticipatory measures which prioritize industrial control and secure water supply. Ultimately, the integration of long-term monitoring data with predictive modeling establishes an evidence-based paradigm for proactive risk management and science-informed policy development.

## Supplementary Information

Below is the link to the electronic supplementary material.

Supplementary Material 1

## Data Availability

The datasets generated and/or analysed during the current study are available from the corresponding author on reasonable request.
