# Supplementary material for: A 24-year integrated assessment of fluoride in surface waters of Jinhua, China: spatiotemporal distribution, risk dynamics, and future projections
Source: Sci Rep. 2026 May 6;16:20924. doi: 10.1038/s41598-026-50957-6 (PMC13338402; doi:10.1038/s41598-026-50957-6)
Supplement: Supplementary file 1 — Supplementary Material 1 [file 41598_2026_50957_MOESM1_ESM.pdf]

Supplementary Materials

The supplementary materials provide detailed supporting information for the study "A 24-year integrated assessment of fluoride in surface waters of Jinhua, China: Spatiotemporal distribution, risk dynamics, and future projections".

Supplementary Table S1. Detailed geographical information and functional characteristics of the 34 water quality monitoring sections across the six studied rivers in the Jinhua Basin.

River Section	Site NO.	Monitoring Section Name	Longitude (°E)	Latitude (°N)	Primary Function and Characteristics
Nanjiang	1	Nanjiang Reservoir Dam	120.35	29.12	Headwater, reference site
	2	Sanjingtou	120.41	29.15	Upstream, forested area
	3	Yanxia	120.48	29.18	Mid-stream, near rural settlement
	4	Fangtang	120.52	29.21	Downstream, before confluence
	5	Nanjiang Bridge	120.55	29.23	Downstream, mixed land use
Dongyang jiang	6	Hengjin Bridge	120.31	29.28	Below Hengjin Reservoir
	7	Xucun	120.38	29.31	Upstream, agricultural
	8	Yidong Bridge	120.44	29.26	Urban section (Dongyang City)
	9	Xingzhong Bridge	120.45	29.25	Urban section, commercial/residential

River Section	Site NO.	Monitoring Section Name	Longitude (°E)	Latitude (°N)	Primary Function and Characteristics
	10	Taxiazhou	120.52	29.24	Downstream, receiving urban runoff
	11	Yangzhai	120.56	29.22	Downstream, mixed industrial
	12	Houqindu	120.59	29.20	Downstream, before confluence
	13	Ditian	120.62	29.18	Downstream, near industrial park
	14	Dongguan Bridge	120.65	29.16	Downstream, entering Jinhua urban area
Wuyijiang	15	Qiangcang	120.18	28.81	Upstream, near Yongkang industrial area
	16	Nanxi Water Plant	120.22	28.85	Water intake, mid-stream
	17	Shiya	120.28	28.88	Mid-stream, dense industrial activity
	18	Tahai	120.32	28.91	Downstream, mixed industrial
	19	Zhangdian	120.35	28.94	Downstream, urban/industrial
	20	Tongqin Bridge	120.38	28.97	Downstream, transportation hub
	21	Baiyangdu	120.42	29.00	Downstream, confluence point
	22	Fancun	120.45	29.03	Downstream, significant pollutant load
	23	Hongwu Bridge	120.48	29.06	Downstream, before confluence with Dongyangjiang
Jinhuajiang	24	Hepan Bridge	119.62	29.10	Upstream, Jinhua urban section
	25	Wucheng Bridge	119.65	29.12	Urban section

River Section	Site NO.	Monitoring Section Name	Longitude (°E)	Latitude (°N)	Primary Function and Characteristics
Lanjiang	26	Shencun	119.68	29.15	Downstream, suburban
	27	Feilong	119.72	29.18	Downstream, before entering Lanjiang
	28	Yanggang	119.48	29.25	Upstream, reference for Lanjiang
	29	Hengshan	119.45	29.28	Mid-stream, near industrial discharge
	30	Nübu	119.42	29.31	Downstream, agricultural and rural
Puyang jiang	31	Jiangjunyan	119.38	29.35	Provincial boundary, downstream control
	32	Jinkengling Reservoir Outlet	119.95	29.51	Headwater, reservoir release
	33	Huangzhai	120.02	29.48	Mid-stream, Pujiang urban area
	34	Shangxianwu	120.08	29.45	Downstream, provincial boundary

Supplementary Table S2. Values, units, and data sources for all exposure parameters used in the health risk assessment model for different river systems and time periods.

Parameter	Symbol	Unit	Value for Lanjiang (2001-2015)	Value for Lanjiang (2016-2024)	Value for Other Rivers	Source
**Fluoride Concentration**	*C*	mg/L	Measured Data	Measured Data	Measured Data	This Study
**Ingestion Rate**	*IR*	L/day	Adults: 2.4; Children : 1.5	Adults: 2.4; Children: 1.5	0.1	32
**Exposure Frequency**	*EF*	days/year	350	15	30	33, Defined by Scenario
**Exposure Duration**	*ED*	years	Adults: 15; Children: 12	9	Adults: 24; Children: 12	34
**Averaging Time**	*AT*	days	ED × 365	ED × 365	ED × 365	34
**Body Weight**	*BW*	kg	Adults: 65; Children: 20	Adults: 65; Children: 20	Adults: 65; Children: 20	35
**Oral Reference Dose**	*RfD*	mg/kg·day	0.06	0.06	0.06	35

Supplementary Table S3. Predictive health risk quotients (HQ) for children and adult populations under the three defined scenarios (Business-as-Usual, Development, Optimization) for the Lanjiang River from 2025 to 2029.

**Scenario**	**Exposure Frequency (days/year)**	**Treatment Factor**	**Predicted HQ (Children)**	**Predicted HQ (Adults)**
Business-as-Usual	15	1.0 (No removal)	0.0170(Mean) 0.0170(Median)	0.0084(Mean) 0.0084(Median)
Development	30	1.0 (No removal)	0.0339(Mean) 0.0339(Median)	0.0167(Mean) 0.0167(Median)
Optimization	7	0.6 (Advanced treatment)	0.0047(Mean) 0.0047(Median)	0.0023(Mean) 0.0023(Median)

Supplementary Table S4. Statistical summary of fluoride concentrations (mg/L) across the six studied river sections. Data are presented as site-year averages. *n*, number of site-year averages; CV, coefficient of variation.

River Section	n	Mean ± SD	Median	Range	CV
Wuyijiang	216	0.893±0.566	0.704	0.135–3.18	0.63
Puyangjiang	72	0.738±0.599	0.464	0.095–2.01	0.81
Jinhuajiang	96	0.617±0.200	0.548	0.380–1.18	0.32
Lanjiang	96	0.449±0.129	0.441	0.226–0.759	0.29
Dongyangjiang	216	0.496±0.242	0.440	0.142–1.38	0.49
Nanjiang	120	0.506±0.241	0.520	0.170–1.54	0.48

Supplementary Table S5. Statistical summary of fluoride concentrations (mg/L) by hydrological season across the entire basin. *n*, number of monthly monitoring data points; CV, coefficient of variation.

Season	n	Mean ± SD	Median	Range	CV
Dry Season	3264	0.701±0.563	0.587	0.075–7.83	0.80
Wet Season	4080	0.613±0.514	0.473	0.073–6.31	0.84
Normal Season	2448	0.606±0.468	0.490	0.079–5.67	0.77

Supplementary Table S6. Spatial contrast in synchronous exceedance frequency of fluoride, COD, and NH_3-N over the 24-year study period.

Group / Station	Number of Monitoring Stations	Total Samples (24-yr)	Samples with Synchronous Exceedance	Synchronous Exceedance Frequency
**Full-Basin**	34	9792	627	6.4%
**Downstream Hotspot Group** (immediately downstream of industrial parks)	4	1152	223	19.4%
**Upstream Background Group**	4	1152	0	0%
**Representative Hotspot Station** Wuyijiang-shiya	1	288	87	30.2%

Notes:

χ^2 test comparing the exceedance frequency between the Downstream Hotspot Group and the Upstream Background Group: $\chi^2(1)=247.87$, $p < 0.001$.

Synchronous exceedance is defined as a sample in which the concentrations of fluoride, COD, and NH_3-N all exceeded their respective thresholds (Fluoride > 1.0 mg/L; COD > 20 mg/L; NH_3-N > 1.0 mg/L, based on Chinese Surface Water Standard Class III).

Supplementary Table S7. Spearman correlation analysis between fluoride and selected water quality parameters (after Bonferroni correction, α'=0.0045).

**Water Quality Parameter**	**Spearman's ρ with Fluoride**	***p*-value (raw)**
Chemical Oxygen Demand (COD)	0.48	<0.001
Ammonia Nitrogen (NH_3-N)	0.43	<0.001
Total Phosphorus (TP)	0.44	<0.001
Total Nitrogen (TN)	0.36	<0.001
Copper (Cu)	0.25	<0.001
Zinc (Zn)	0.24	<0.001
Cadmium (Cd)	0.25	<0.001
Chromium (VI) (Cr^{6+})	0.26	<0.001
Cyanide (CN^-)	0.23	<0.001
Volatile Phenol (VPh)	0.42	<0.001
Total Petroleum Hydrocarbons (TPH)	0.26	<0.001

Supplementary Table 8. Results of the Mann-Kendall trend test for basin-wide fluoride concentrations in Jinhua.

Period	Mann-Kendall τ	Z statistic	p-value	95% Confidence Interval	Trend Significance (α=0.05)
Full Period 2001-2024	-0.594	-4.05	< 0.001	[-0.880, -0.308]	Significant trend
Phase 1: 2001-2013	-0.372	-1.71	0.087	[-0.783, 0.039]	No-significant trend
Phase 2: 2014-2024	0.145	0.55	0.58	[-0.307, 0.598]	No-significant trend

Supplementary Table S9. Results of the Mann-Kendall trend test for fluoride concentrations in the Lanjiang River.

Period	Mann-Kendall τ	Z statistic	p-value	95% Confidence Interval	Trend Significance (α=0.05)
Full Period 2001-2024	-0.257	-1.76	0.079	[-0.545, 0.032]	No-significant trend
Phase 1: 2001-2013	-0.282	-1.28	0.20	[-0.694, 0.130]	No-significant trend
Phase 2: 2014-2024	0.127	0.47	0.64	[-0.330, 0.585]	No-significant trend

Supplementary Table S10. Results of the Mann-Kendall trend test for health risk (Hazard Quotient, HQ) in the Lanjiang River and Other rivers.

Object	Period	Mann-Kendall τ	Z statistic	p-value	95% Confidence Interval	Trend Significance (α=0.05)
Lanjiang River	Full Period 2001-2024	-0.434	-2.92	0.004	[-0.725, -0.143]	significant trend
Lanjiang River	Phase 1: 2001-2015	-0.238	-1.44	0.15	[-0.560, 0.084]	No-significant trend
Lanjiang River	Phase 2: 2016-2024	0.167	0.54	0.59	[-0.346, 0.679]	No-significant trend
Other Rivers	Full Period 2001-2024	-0.362	-2.46	0.014	[-0.648, -0.077]	significant trend

Supplementary Table S11. Key parameters, performance metrics, and residual diagnostics for the Holt-Winters and ARIMA(1,1,1) time-series models used for fluoride concentration forecasting. MAE, Mean Absolute Error.

**Parameter / Metric**	**Holt-Winters (Additive)**	**ARIMA(1,1,1)**
**Smoothing Parameters / Coefficients**	Level (α) = 0.62<br>Trend (β) = 0.05<br>Seasonal (γ) = 0.15	AR Lag1 = –0.41<br>MA Lag1 = 0.72
**Model Fit**	R^2 = 0.83<br>MAE = 0.029 mg/L	R^2 = 0.79<br>MAE = 0.034 mg/L
**Residual Diagnostics**	—	Ljung–Box test: p = 0.08 (supports model adequacy)
**Combination Forecast Weight**	0.55	0.45

Supplementary Table S12. Forecasted annual mean fluoride concentrations (mg/L) for the Lanjiang River and other river systems from 2025 to 2029, generated by the Holt-Winters, ARIMA(1,1,1), and the final coupled forecasting model.

**Year**	**Holt-Winters**		**ARIMA(1,1,1)**		**Coupled Model**	
	Other Rivers	Lanjiang River	Other Rivers	Lanjiang River	Other Rivers	Lanjiang River
2025	0.432	0.328	0.425	0.330	0.429	0.329
2026	0.428	0.326	0.435	0.335	0.431	0.330
2027	0.425	0.323	0.443	0.339	0.433	0.330
2028	0.421	0.321	0.450	0.343	0.434	0.331
2029	0.418	0.319	0.458	0.346	0.436	0.331

Notes: The Holt-Winters model predicts a gradual decline in concentration, whereas the ARIMA model forecasts a moderate upward trend. This discrepancy stems from their distinct approaches to handling trend and volatility components in the time series.
